# The sculpting of somatic mutational landscapes by evolutionary forces and their impacts on aging‐related disease

**DOI:** 10.1002/1878-0261.13275

**Published:** 2022-06-29

**Authors:** Fabio Marongiu, James DeGregori

**Affiliations:** ^1^ Department of Biochemistry and Molecular Genetics University of Colorado Anschutz Medical Campus Aurora CO USA; ^2^ Department of Biomedical Sciences, Section of Pathology University of Cagliari Italy; ^3^ University of Colorado Comprehensive Cancer Center University of Colorado Anschutz Medical Campus Aurora CO USA

**Keywords:** aging, clonal hematopoiesis, life‐history theory, NOTCH1, p53, somatic evolution

## Abstract

Aging represents the major risk factor for the development of cancer and many other diseases. Recent findings show that normal tissues become riddled with expanded clones that are frequently driven by cancer‐associated mutations in an aging‐dependent fashion. Additional studies show how aged tissue microenvironments promote the initiation and progression of malignancies, while young healthy tissues actively suppress the outgrowth of malignant clones. Here, we discuss conserved mechanisms that eliminate poorly functioning or potentially malignant cells from our tissues to maintain organismal health and fitness. Natural selection acts to preserve tissue function and prevent disease to maximize reproductive success but these mechanisms wane as reproduction becomes less likely. The ensuing age‐dependent tissue decline can impact the shape and direction of clonal somatic evolution, with lifestyle and exposures influencing its pace and intensity. We also consider how aging‐ and exposure‐dependent clonal expansions of “oncogenic” mutations might both increase cancer risk late in life and contribute to tissue decline and non‐malignant disease. Still, we can marvel at the ability of our bodies to avoid cancers and other diseases despite the accumulation of billions of cells with cancer‐associated mutations.

## Graphical abstract

Pathogenic clonal expansions can promote and be promoted by inflammation, and contribute to multiple diseases of aging. While these clones can sometimes directly contribute to malignant disease, as clearly demonstrated for leukemias with clonal hematopoiesis mutations, evidence also reveals how clonal expansions can contribute indirectly to cancers and non‐malignant diseases such as through the promotion of inflammation.

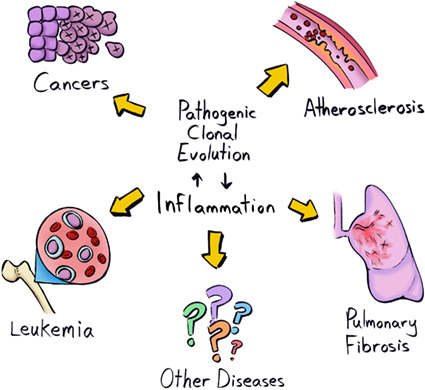

**Abbreviations**
AML: acute myeloid leukemia
APC: adenomatous polyposis coli protein (APC) regulator of WNT signaling pathway
ARCH: age‐related clonal hematopoiesis
CHIP: clonal hematopoiesis of indeterminant potential
COL17A1: collagen type XVII alpha 1 chain
CVD: cardiovascular disease
CXCL14: C‐X‐C motif chemokine ligand 14
DNMT3A: DNA methyltransferase 3 alpha
ECM: extracellular matrix
EDAC: epithelial defense against cancer
ERBB2: Erb‐B2 receptor tyrosine kinase 2
ERBB3: Erb‐B2 receptor tyrosine kinase 3
FGF3: fibroblast growth factor 3
FOXO1: forkhead box O1
HRAS: Harvey rat sarcoma proto‐oncogene, GTPase
HSC: hematopoietic stem cells
HSP90: heat shock protein 90
HSPC: hematopoietic stem and progenitor cells
ILBP2: UL16‐binding protein 2
IR: ionizing radiation
KMT2D: lysine methyltransferase 2D
KRAS: Kirsten rat sarcoma proto‐oncogene, GTPase
MICA: MHC class I polypeptide‐related sequence A
MYC: MYC proto‐oncogene, BHLH transcription factor
NFKBIZ: NFKB inhibitor zeta
NKG2D: killer cell lectin like receptor K1
NOTCH1: notch receptor 1
NRAS: neuroblastoma RAS proto‐oncogene, GTPase
PASP: p21‐activated secretory phenotype
PIK3CA: phosphatidylinositol‐4,5‐bisphosphate 3‐kinase catalytic subunit alpha
SASP: senescence‐associated secretory phenotype
TET2: tet methylcytosine dioxygenase 2
TP53: tumor protein P53

## 1 Introduction

Cancer is thankfully relatively rare in humans before the age of 50 but the odds of developing cancer rise exponentially later in life [1]. The risk of many other diseases (such as heart disease, kidney failure, and neurodegeneration)—and of course death—also rises exponentially later in life, often with similar kinetics [2]. Even deaths from infectious agents rise exponentially later in life, due in large part to declining immune function, as has become painfully apparent during the COVID19 pandemic [3]. The same associations are seen in our domesticated pets, in laboratory animals, such as mice, and in animals maintained in zoos [4]. While senescent animals (who are undergoing aging‐dependent tissue dysfunction) are more rare, given the much higher extrinsic mortality of animals in the wild, studies have shown that cancer frequency increases specifically at older ages [5, 6, 7].

Studies over the last decade have revealed the striking presence of cancer‐associated mutations in what appear to be healthy tissues [8], as first shown for *TET2* mutations in the blood of elderly people [9]. Additional studies have reported the presence of numerous leukemia‐associated mutations in the blood of healthy individuals, and an exponential rise in the fraction of leukocytes occupied by these mutant clones at older ages (starting at ∼40 years; reviewed in [10, 11, 12]). When these clones are detected above a certain threshold (typically, 2% of nucleated blood cells), this condition is called Clonal Hematopoiesis of Indeterminant Potential (CHIP) (Box 1) or Age‐Related Clonal Hematopoiesis (ARCH)—we refer to it as CHIP here. CHIP is associated with substantial increases in the risk of leukemia and of other cancers, as well as with other aging‐related diseases, such as heart disease, lung diseases (such as chronic obstructive pulmonary disease), frailty, and overall mortality [11, 12, 13]. Recent studies have shown that tissues throughout our body become riddled with clones bearing cancer‐associated mutations, with their frequency increasing as we age and varying by tissue (reviewed in refs [8, 14, 15]). In fact, epithelial layers of the esophagus, endometrium, and skin become dominated by mutant clones in older individuals, with additional but differential contributions to colon, bladder, lung, liver, or neurons [8]. Strikingly, it appears that older individuals possess over 100 billion cells with cancer‐associated mutations even though many tissues have yet to be analyzed and clone detection is limited by the sensitivity and specificity of the sequencing methods used [8].

Box 1 **Glossary**

**Antagonistic pleiotropy:** according to this evolutionary theory, some genetic variants can have multiple effects (pleiotropy), and in some cases, the effects can be antagonistic (one is beneficial, another one is detrimental for the organism). In the context of aging, specific variants can be positively selected because they increase overall fitness, such as by increasing reproductive output and/or robustness in youth, while also contributing to aging or disease risks later in life.

**Clonal fixation:** a phenomenon that occurs when a specific mutation becomes homogeneously present in a cell population, following the clonal expansion of a mutated cell. In the context of colonic crypts, clonal fixation occurs when a mutated stem cell slowly expands to replace neighbor stem cells so that the entire crypt eventually has that particular mutation.

**Clonal hematopoiesis (CHIP)**: an age‐associated phenomenon in which the blood of healthy individuals exhibits a fraction of leukocyte clones (typically defined as above 2%) bearing leukemia‐associated mutations. This condition is associated with an increased risk of leukemia and other aging‐related diseases.

**Driver mutation:** a mutation within a gene that confers a selective growth advantage (greater somatic fitness), and thus has the potential to contribute to cancer development.

**Dysbiosis:** an imbalance between different types of microorganisms normally present in an individual's microflora (e.g., in the gut) that can contribute to a range of diseases.

**Life‐History theory**: a theory to study how natural selection leads to organismal traits such as size, age of maturity, number of offspring, life span, etc., in order to maximize reproductive success, taking into account how environmental and intrinsic factors can selectively affect survival and reproduction.

**Somatic fitness**: the fitness of cells that make up the soma (the bulk of non‐reproductive cells in our bodies), measurable as the ability of a particular cellular genotype or epigenotype to contribute to subsequent cellular generations in that tissue. Somatic mutations or epigenetic changes that increase or decrease somatic fitness will increase or decrease (respectively) the odds that that clone continues to contribute to the tissue over time.

**Stabilizing selection:** a type of natural selection in which genetic diversity decreases as the population stabilizes on particular non‐extreme trait values.

So, what are the implications of this astounding observation? In this review, we discuss the potential impacts of this age‐related increase in cancer‐associated mutations for disease risk and for declining tissue health. We discuss why somatic evolution becomes more prevalent in old age and review the many mechanisms that animals have evolved to keep somatic evolution in check to maximize organismal fitness. We also argue that somatic evolution is critical for keeping pre‐malignant (oncogenically initiated) cells in check and for limiting the expansion or pathogenicity of more‐advanced malignancies. We discuss how eliminating malfunctioning cells from tissues is important for maintaining functionality, and thus youthful robustness and individual fitness. As such, we propose that maintaining healthy (“youthful”) tissues is vitally important for delaying tissue decline (tissue senescence) and the increased risk of disease. We place our discussions within the context of Life History theory (Box 1) and the understanding that natural selection favors strategies that invest in the soma to the extent that maximizes reproductive success [16]. This perspective frames our understanding of how somatic evolution can be disfavored, ignored, or even favored, depending on how it impacts tissue health and disease risk, and thus animal fitness, and how the limitations of these strategies can contribute to disease risk, particularly as we age.

## 2 Somatic evolution—from neutral to damaging and possibly beneficial

**Fig. 1:**
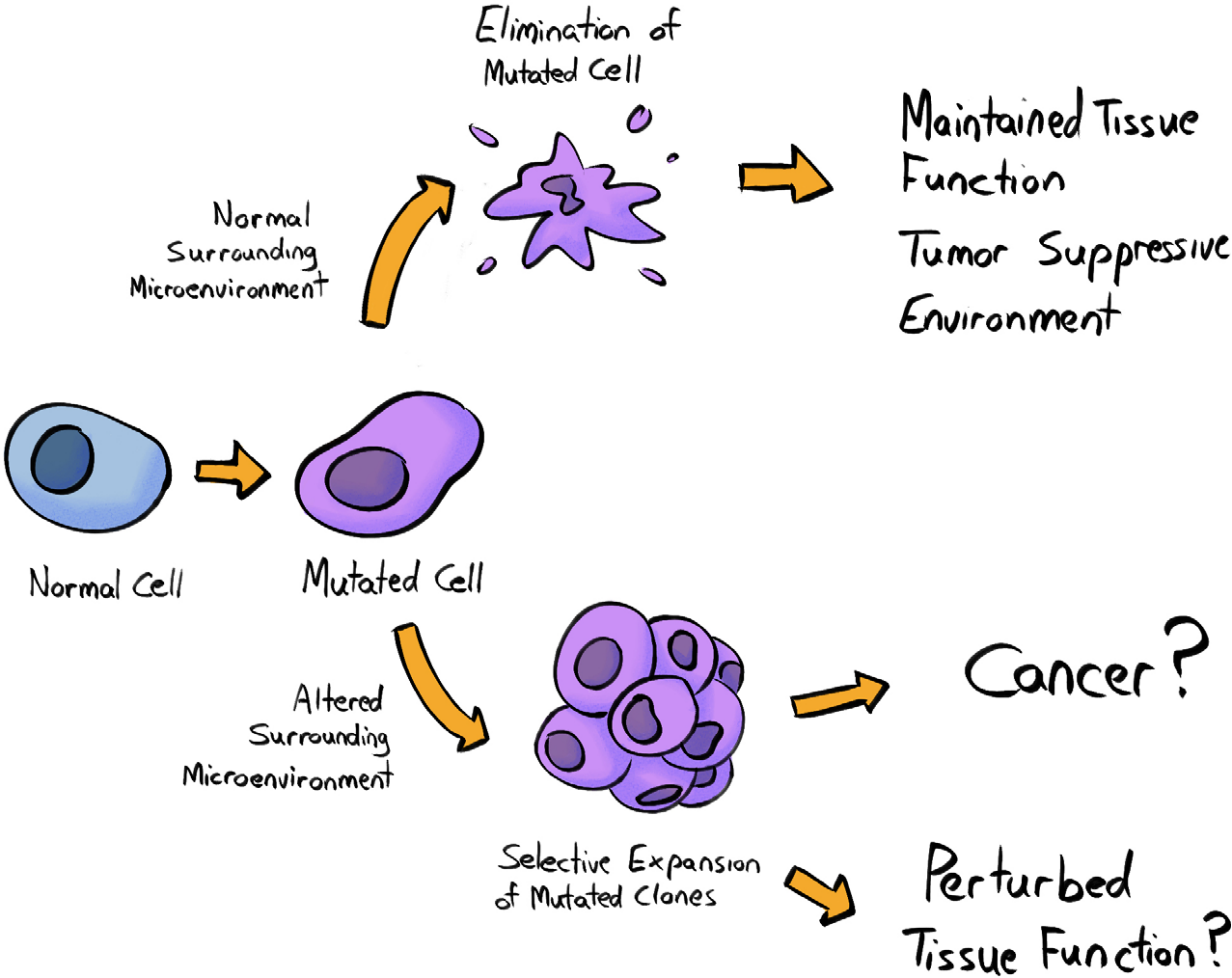
Clonal evolution of somatic cells is influenced by cues from the surrounding microenvironment. In the context of a normal tissue landscape, cells harboring specific mutations are likely eliminated, or their proliferation is restrained, thus contributing to the maintenance of tissue function and a tumor‐suppressive environment. On the other hand, the expansion of the same mutated clone might be promoted in the context of an altered surrounding microenvironment. The accumulation of clones harboring genetic and/or epigenetic changes might directly contribute to oncogenesis and/or to progressively establishing a tissue landscape with perturbed function. Note that this figure is likely oversimplified, and should not be interpreted to indicate that a mutant clone will *never* expand in a healthy tissue microenvironment, but that altered microenvironments should promote the expansion of clones with mutations that provide an adaptation to that environment. [Colour figure can be viewed at wileyonlinelibrary.com]

Somatic evolution encompasses the changes in the clonal makeup of tissues, facilitated by mutations and epigenetic changes that accumulate in cells with age as well as tissue microenvironmental changes that influence somatic cell fitness (Fig. 1). A key first principle of somatic evolution is that natural selection acts to maximize reproductive success, and that soma have evolved to maximize the chances of the germline being passed to subsequent generations. We should also recognize that perfection (or any optimum) is never achieved, as there are limitations to biological machinery and mechanisms (e.g., mutation rates could never be zero) and that trade‐offs are involved (such as energetic or other costs that exceed the provided benefits). With this in mind, let us reframe our understanding of somatic evolution from an evolutionary perspective: how has natural selection over millions of years sculpted developmental and tissue maintenance programs that minimize the fitness costs of somatic evolution, and perhaps even maximize the benefits?

### 2.1 The life‐history framework

A life‐history perspective is of vital importance for understanding why we age and why the risk of many diseases, including cancers, increases in our later years. While modern humans in industrialized nations experience long lifespans, for most of our evolutionary history, we rarely lived and/or reproduced into our “golden years.” For example, the Native American people who buried their dead in the Dickson Mounds (in current‐day Illinois) around the first millennium AD rarely lived past 50 [17]. Similarly, the Meinarti people of the 6‐7th century, of what is now northern Sudan or southern Egypt, rarely survived past 50 years of age [18]. Additional fossil evidence indicates that only ∼10% of our African ancestors 50 000 years ago lived to 40, and < 5% to 50 [19, 20]. For much of human history, each year of life brought a reasonable chance of not surviving to the next; thus, one's odds of successful reproduction declined with age [21]. Of course, survival into what is now considered to be old age is also rare for other animals [22]. For example, studies show that wild mice do not survive past 1 year of age, and most are dead by 6 months [23], well before most manifestations of aging or cancer development can become evident (mice live for up to 2–4 years in laboratories and have an increased risk of cancer from 18 months of age) [24]. The reality is that natural selection is unlikely to heavily invest in maintaining the soma beyond the age of reproduction, especially given the extrinsic factors that limit survival, such as predation, starvation, cold, and disease. It is important to point out, however, that somatic maintenance is not suddenly turned off—instead, the investment wanes over time as the likelihood of contributing to reproduction declines. As one example, parental and grandparental care in human populations likely extends the pressure for somatic maintenance well beyond the age when an individual is still reproductive [25].

Thus, aging, with its associated increased risks of disease and death, results from a waning investment in somatic maintenance. So, what does this mean for somatic evolution and for the increased incidence of detectable clones in our tissues in later life, many of which are driven by cancer‐associated mutations? We first need to consider the impacts of these expanding clones on tissue function and organismal health, which fall into three categories: neutral, damaging, or advantageous (from the *organism's* perspective). At present, despite the presence of hundreds of mutation‐associated expanding clones in our tissues [8], we do not know which of these clones fall into the neutral category. We speculate that these clones have a minimal‐to‐no impact on tissue function and do not confer a significant risk of malignancy or other diseases. If a mutation that marks a clone is not found in cancers arising from this tissue at a rate higher than its presence in normal tissue, it is likely to be neutral or nearly neutral in its impact on carcinogenesis. As discussed below, the tissue microenvironment can also minimize the phenotypic manifestations potentially induced by mutations, even oncogenic mutations [26, 27]. Thus, a normal context can have a normalizing influence on mutated clones, which can mitigate the clones' potential negative consequences. Nonetheless, clones can reduce tissue function without contributing to a malignancy. Important but unanswered questions remain. For example, can a mutation confer a selective advantage to a clone in a tissue without impacting the functional attributes of the tissue's cells? Does the clone, including its more differentiated progeny, still function as it should in the tissue? Positive selection for a mutation may still lead to a clonal expansion that is neutral with respect to its impact on the organism. We currently lack answers to these questions, which require us to recreate identified mutations in expanded clones in tissues of model organisms and then analyze their impacts on the tissue (and not just on malignant progression).

### 2.2 The impact of expanding clones on animal fitness

Expanded clones can negatively impact tissue function and thus individual fitness by either contributing to a malignancy or by otherwise reducing tissue function. By fitness, we are referring to the ability of an individual to survive and reproduce. Healthy tissues in youth are key to avoiding predation, to procuring resources like food, to securing a mate, and surviving uncertain environments. Natural selection has acted over millions of years to engender tissue developmental and maintenance programs that maximize fitness. We need to consider somatic evolution from this perspective.

#### 2.2.1 The role of CHIP in cancer and disease

**Fig. 2:**
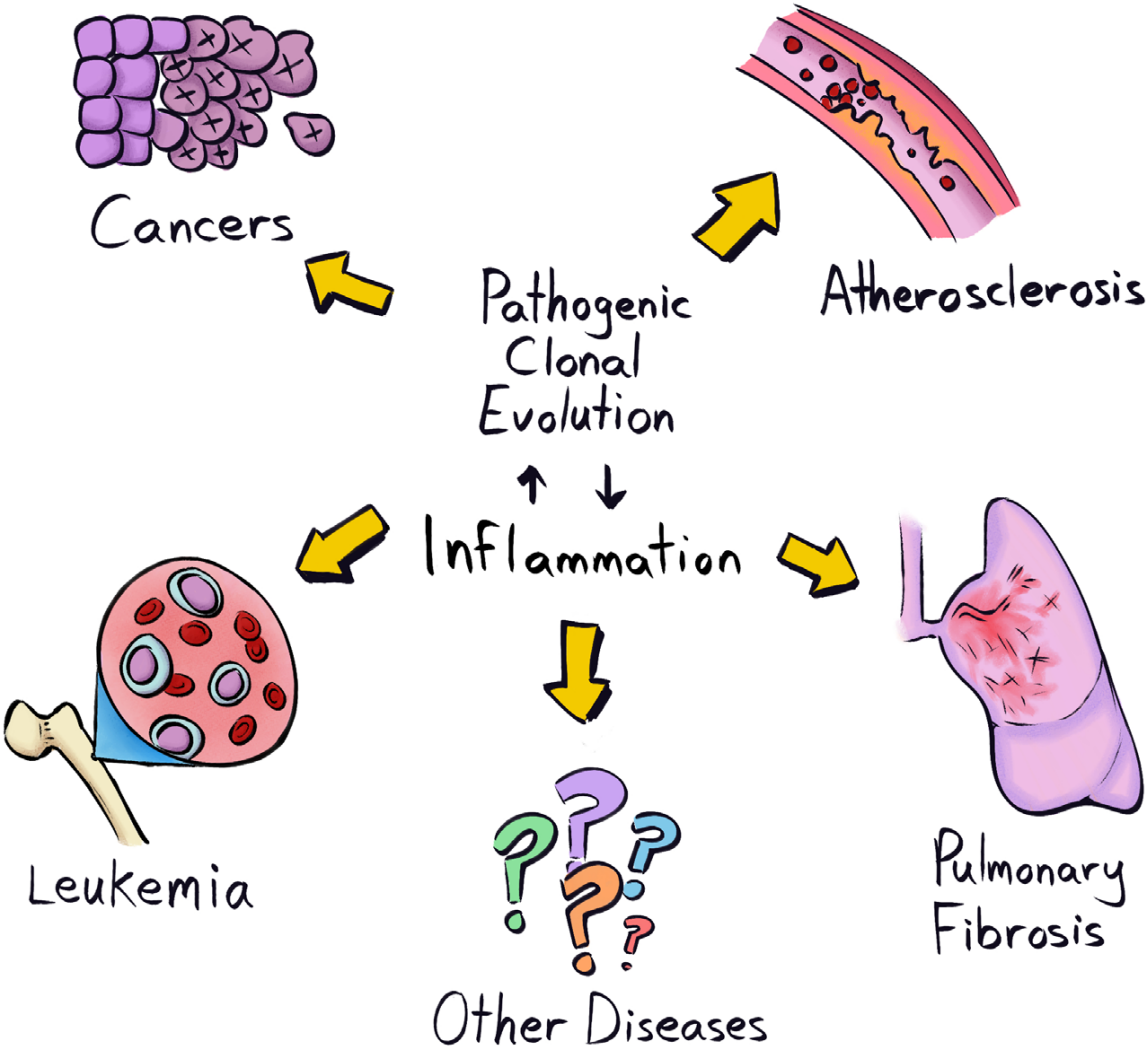
Clonal evolution and disease. Pathogenic clonal expansions can promote and be promoted by inflammation, and contribute to multiple diseases of aging. While these clones can sometimes directly contribute to malignant disease, as clearly demonstrated for leukemias with clonal hematopoiesis mutations (and also likely the case for mutations in solid tissues, such as in TP53 or PIK3CA), evidence also reveals how clonal expansions can contribute indirectly to cancers and non‐malignant diseases (like cardiovascular disease, CVD, which is associated with CHIP) such as through the promotion of inflammation. Finally, although still poorly established, emerging evidence suggests direct roles for clonal expansions in non‐malignant disease such as for fibroblasts in pulmonary fibrosis or for vascular smooth muscle cells in CVD [51]. [Colour figure can be viewed at wileyonlinelibrary.com]

Expanded clones that contribute to disease risk can do so by acting as cancer‐initiating events and/or by changing the tissue microenvironment. Such associations have been most clearly shown for CHIP and for the subsequent development of acute myeloid leukemia (AML). CHIP is frequently characterized by mutations that are known to cause leukemias. In blood samples of individuals obtained years before the onset of AML, mutations were identified in the CHIP clone that were then found in the subsequently developed AML [28, 29, 30]. That said, we are unaware of systematic analyses of the *fraction* of AML cases where CHIP has been previously detected for which the identified CHIP mutation was present in the AML. Still, it is notable that CHIP is associated with a substantial increase in the risk of all cancers (most of which will be carcinomas) [10, 11, 12]. In such cases, the CHIP clone is clearly not contributing directly to the cancer clone. We propose two possible explanations. First, the CHIP clone could promote oncogenesis by changing the tissue microenvironment. For example, CHIP clones marked by *TET2* mutations lead to the production of mature myeloid cells that produce inflammatory cytokines [11], and inflammation is associated with cancer evolution. Second, CHIP could be a marker of an aging or damaged soma, and thus could represent a common consequence of this somatic decline (along with increased cancer risk). For example, cigarette smoking is associated with an increased risk of CHIP, lung cancer, heart disease, emphysema, and other maladies [2, 12, 31]. But CHIP is unlikely to be responsible for all of these other consequences of smoking, although it may help to amplify some of their negative consequences by increasing inflammation. Finally, studies have shown how inflammation (such as via infections) can select for CHIP mutations, including in *TET2* [32, 33, 34], indicating the role of a positive feedback loop (Fig. 2).

#### 2.2.2 Mutant clones in solid tissues and disease

Clones in solid tissues are often driven by cancer‐associated mutations but we know less about how these clones contribute to, or are a risk factor for, cancer. In part, their lack of association with disease likely reflects that fewer samples have been analyzed in studies of clonal architecture in solid tissue. These studies typically involve at most dozens of samples [8], as opposed to the many tens of thousands of samples analyzed for CHIP [10, 11, 12]. Still, it is notable that a fraction of detected clones bear oncogenic mutations that are common in cancers of that same tissue. Studies of the esophagus provide an informative example. The most common mutations that appear to drive clonal expansions in this tissue are in the *NOTCH1* gene, and these mutations are very similar to those found in human cancers [35, 36]. These *NOTCH1* mutations are present in well over half of esophageal epithelial cells at older ages (and also become prevalent in the skin epithelium [35, 37], at least in the studied cohorts with European ancestry). The frequency of these mutations is therefore substantially higher in all epithelial cells than in human esophageal cancers (10–15% of which bear *NOTCH1* mutations). Thus, cells with *NOTCH1* mutations give rise to cancer at a rate that is substantially *less* than would be expected by chance. In contrast, mutations in the *TP53* gene are much less frequent in the esophageal epithelium, and are mostly present in older subjects. *TP53* mutations are present in ∼ 90% of esophageal cancers, raising the question as to whether these mutations in non‐malignant esophageal cells is a risk factor for carcinoma development. While causative associations cannot at present be made, these clonally expanded *TP53* mutations are known to contribute to carcinogenesis, such that their presence “at the scene of the crime” makes them strong suspects. Similarly, mutations in the *PIK3CA* gene are detected in the endometrium, and can contribute to endometrial cancers [38]. While these examples might provide insight into the earliest events in malignant progression, we need to consider that most of these mutation‐bearing cells will never contribute to a cancer. Indeed, most mutations associated with clonal expansions in our tissues, while often identifiable in some cancer(s), are most often variants that rarely recur across cancers (see table S1 in ref. [8]). While more research will be necessary to statistically contrast mutational spectra in young and old individuals (with a focus on malignant potential), we would speculate that natural selection has led to tissues that are particularly good at preventing clonal expansion driven by malignant mutations.

Accordingly, we should consider examples of oncogenic driver mutations (Box 1) associated with cancer in a particular tissue that are rarely detected in non‐malignant states. Colonic crypts provide a good example. These crypts are known to drift to clonal fixation (Box 1) every few years. As such, sequencing can identify clonal mutations that come to occupy the full crypt. Notably, while mutations in *APC, KRAS,* and *TP53* are present in most colon cancers, they are almost never found in normal human colonic crypts [39]. Of note, *APC* mutations are known to initiate colon carcinogenesis [40]. In contrast, known driver mutations in *ERBB2* and *ERBB3* are common in normal crypts, but are rare in colon cancers. While only ∼ 1% of crypts contain driver mutations common in colorectal cancer [39], a recent re‐analyses of the data from this study revealed that nearly all crypts contain at least one mutation previously associated with any human cancer, which is far greater than would be expected by chance [8]. Again, most of these mutations are rarely observed in colon cancers (and indeed are not highly prevalent in other human cancers). Perhaps, mutations in key drivers of colon cancers (such as in the *APC*, *KRAS,* and *TP53* genes) are strongly selected against in crypt stem cells. That said, the modeling of *APC* mutations in the mouse intestinal crypt indicates that *APC* mutations confer an advantage to intestinal stem cells [41, 42, 43]. Additional studies are needed to resolve this conundrum.

While the focus of somatic evolution is typically on oncogenesis, some mutations perturb tissue function, by impairing differentiation, reducing functionality, and by negatively impacting neighboring cells and tissues. We have already discussed how CHIP (most studied for clones driven by TET2 mutations) can alter the function of myeloid cells, leading to heightened inflammation and disease risks [11], with a notable association with cardiovascular disease (CVD). In mouse models, altered hematopoiesis caused by *TET2‐* and *DNMT3A*‐mutation can potentiate CVD, coinciding with the enhanced production of inflammatory cytokines from mutant myeloid cells [44, 45, 46, 47]. In epithelial cells, as previously discussed, clones form contiguous patches, as seen in the skin, esophagus, colon, and endometrium [35, 36, 37, 38, 39] . Many clonally expanded mutations in these tissues impact key signaling pathways known to regulate the differentiation, function, and survival of these cells, such as mutations in the Notch, PI3K/Ras, and p53 pathways and in key regulators of histone methylation (such as *KMT2D*). Do these clones impact epithelial function or does the overall tissue architecture exert a normalizing effect that suppresses negative outcomes? At present, we do not know. Still, given that several of these tissues (skin, airways, digestive track) perform critically important barrier functions, their perturbation by mutant patches could produce systemic effects. For example, the increased influx of bacteria and their products could lead to systemic inflammation. Intestinal permeability also increases in old age, promoting systemic inflammation [48, 49]. Future studies will need to determine whether the aging‐related expansion of clones with oncogenic mutations in the epithelia contributes to this loss of barrier function, leading to a major contributor to human aging—inflammation. Notably, not all damaging evolution is restricted in its impact to the soma. With increasing frequency in older males, *FGFR3* and other mutations are selected for in seminiferous tubules and can negatively impact fitness in offspring [50].

Finally, as recently reviewed by Majeti and colleagues [51], clonal expansions are *directly* associated with diseased tissues (beyond cancers), including atherosclerosis and multiple fibrotic diseases, and common idiopathic pulmonary fibrosis. Additional studies are needed to establish the extent to which clonal expansions directly contribute to these diseased tissues, the mutations or epigenetic changes associated with these expansions, and what, if any, contexts drive selection for these (epi)genetic changes. Notably, all of these diseases are associated with inflammation, and we can speculate that a positive reinforcement loop exists in which inflammation and clonal expansion each promote the other (Fig. 2).

#### 2.2.3 Expanded clones and trade‐offs

While somewhat more speculative, some expanded clones may be driven by mutations that reduce the risks of cancer. As mentioned above, the frequency of *NOTCH1* mutant clones in the esophagus outpaces their frequency in associated carcinomas, indicating selection *against NOTCH1* disruption during cancer evolution. Perhaps, clones with *NOTCH1* mutations confer a more benign alternative to more malignant mutations, and tissue structures that select for such clones as we age may have been favored by natural selection in order to delay cancer development [52]. Another notable example is the high prevalence of interleukin‐17 (IL‐17) and Toll‐like receptor pathway mutations such as in *NFKBIZ*, in patients with ulcerative colitis [53, 54, 55]. There is clear evidence of strong positive selection for *NFKBIZ* mutations in the colonic epithelium, and a *NFKBIZ* mutation clone can expand to encompass a substantial fraction of the tissue *specifically* in individuals with colitis. IL‐17 pathway mutations confer resistance to the pro‐apoptotic effects of IL‐17 signaling associated with colitis. Notably, cells with *NFKBIZ* mutations are almost never found in colon cancers, and mice with *NFKBIZ* genetic disruption are resistant to the induction of colon adenocarcinomas [53], indicating that such expansions impede carcinogenesis. Conversely, others have argued that IL‐17 pathway disruption might contribute to dysbiosis (Box 1) [55]. In the liver, cirrhosis drives the selection of clones that have mutations adapted to this context; their presence appears to improve liver function and regeneration [56]. On the other hand, clonal expansions in *FOXO1* are also evident in the livers of those with chronic liver disease, and these mutant clones are expected to negatively impact insulin‐dependent glucose and lipid metabolism [57]. Thus, clonal expansions likely have both positive and negative health consequences. Finally, trade‐offs might have evolved in the maintenance of tissue function vs. cancer risk. For example, a recent study [58] has shown that pancreatitis‐mediated epigenetic remodeling promotes the selection of oncogenic *KRAS* mutations, leading to reduced tissue damage in future bouts of pancreatitis, while at the same time increasing the risks of pancreatic cancer (which in humans mostly occurs in old age).

In all, given the strong selective pressure to maintain tissue health and to prevent cancers through reproductive ages, together with the observation that expanded clones occur at younger adult ages [8] and that cancers are rare at these ages, we can make two predictions: (1) that clones that reduce tissue function or promote malignancy will rise in frequency later in life when reproductive success becomes progressively lower; and (2) that clones that are more neutral or even beneficial to tissues will arise earlier in life, as natural selection has not acted to sculpt tissue programs that prevent the advent of these clones and may even have sculpted tissue landscapes that favor the beneficial ones. Given the importance of avoiding detrimental somatic evolution to the extent that maximizes individual fitness, we will discuss how aging can alter tissue landscapes to facilitate damaging somatic evolution.

## 3 Altered adaptive landscapes in aging or damaged tissues

Aging is a complex process characterized by the progressive accumulation of structural and functional changes at the cell, tissue, and organ level [59]. Tissue landscapes evolve over time, determining the shape and direction of somatic evolution [60]. Lifestyle and disease can drastically influence the pace and intensity at which these landscapes change and thus affect the trajectories of somatic evolution [61]. Indeed, the selection of specific oncogenic events depends on context. Several studies have now shown that the surrounding tissue microenvironment plays a major role in determining the fate of altered cells [60, 62, 63, 64, 65]. While young tissues are normally tumor suppressive and disfavor the clonal expansion of initiated cells (i.e., under stabilizing selection, Box 1), in aged tissue the widespread reduction of fitness might provide an adaptive landscape that favors their selective proliferation (i.e., positive selection).

As a demonstration of context‐dependent selection, mouse hematopoietic stem and progenitor cells (HSPCs) that bear oncogenic mutations are not selected for when transplanted into the bone marrow environment of a younger mouse, while the same oncogenically initiated HSPCs selectively expand in the hematopoietic environment of an aged mouse [66, 67, 68]. Activating *KRAS* mutations in the mouse lung also lead to more frequent and larger tumors in aged compared to young mice [69]. In another example, the liver microenvironment of old rats is clonogenic for both normal and preneoplastic transplanted hepatocytes, compared to the liver of younger rats, where the proliferation of these same cells is limited following their transplantation [70, 71]. These results reveal how an aged tissue microenvironment allows for, and even promotes, the competitive expansion of fitter cell clones, whether bearing oncogenic mutations or not [72]. A functional role of the aged microenvironment in the development of cancer has been described in several tissues, including in the skin [73], ovary [74], mammary gland [75], prostate [76], and colon [64].

Although the emergence of mutated cells as we age is unavoidable, we can influence their selection by modulating the tissue microenvironments where they reside. The increased burden of neoplastic disease in the old can be partly ascribed to the widespread, low‐grade inflammation, commonly observed during aging [66, 69, 77, 78], although the mechanisms behind this association are not fully elucidated. Notably, inhibiting inflammation prevents the aging‐associated selection of oncogenic mutations in the mouse hematopoietic system [66], highlighting the key role of the microenvironment in dictating evolutionary trajectories. Similarly, caloric restriction and time‐restricted feeding both reduce the emergence and expansion of pre‐neoplastic nodules in the liver, by delaying the onset of the neoplastic‐prone tissue landscape that is typical of aging [79, 80]. In this experimental setting, both inflammatory and cell competition mechanisms are involved [81]. In fact, when normal young hepatocytes were infused into the livers of rats with induced hepatocellular carcinoma, the selective proliferation of “healthy” cells with a higher competitive fitness was able to delay the growth of pre‐neoplastic nodules and to limit their progression to cancer [82]. This highlights once again the importance of the surrounding microenvironment for clonal evolution in tissues.

It is well established that major avoidable risk factors, such as smoking, alcohol consumption, and UV light exposure, drastically increase the risk of cancer, although the onset of disease remains strongly age‐dependent [1, 83]. In addition to the direct mutagenic impact that such exposures have on our cells, it is reasonable to hypothesize that the ensuing chronic and cumulative damage that affects the bulk of the tissue sets the stage for the selective expansion of fitter clones, including those at risk of transformation. In a recent study on the effect of smoking on the bronchial epithelium, at least 25% of bronchial epithelial cells carried oncogenic driver mutations, vs. 4–14% in non‐smoking middle‐aged individuals; each cell also had a significantly higher number of mutations in smokers ([84]; see also [85]). Intriguingly, a small population of lung epithelial progenitors was found to be spared from smoking‐promoted mutagenic events that could repopulate the epithelium upon smoking cessation [84]. This suggests that the selective expansion of mutated clones is highly dependent on the widespread toxicity that smoking exerts on the tissue landscape and that, upon elimination of the toxic insult, normal progenitors selectively repopulate the lung.

Similarly, studies conducted on the esophageal epithelium of transgenic mice show that rare *Tp53* mutant progenitors are normally present in the mucosal compartment. However, their selective proliferation over wild‐type progenitors only occurs after their exposure to low‐dose ionizing radiation [86]. Most importantly, upon administration of an antioxidant to prevent oxidative damage, the fitness of wild‐type progenitor cells was restored, thus limiting the expansion of *TP53* mutant clones [86]. Similar observations have been reported for UV light exposure in human skin [87], highlighting the possible role of the photoaged tissue microenvironment in the pathogenesis of melanoma [73].

In all, these studies reveal that youthful tissues can suppress oncogenesis and cancer development, while tissue impairment with age or damage can promote tumorigenesis. In the next section, we will delve into the mechanisms that we and other animals have evolved to eliminate mutated cells which could otherwise disrupt tissue function or contribute to malignant evolution.

## 4 Evolved mechanisms to deal with somatically mutated clones

Complex organisms, such as humans, consist of an enormous number of cells that are repeatedly exposed to various insults and go through numerous rounds of replication. Although each cell in our body, on average, accumulates about 20 mutations per year [88], and each adult body consists of ∼3 trillion nucleated cells [89], most individuals live cancer‐free for at least half a century.

**Fig. 3:**
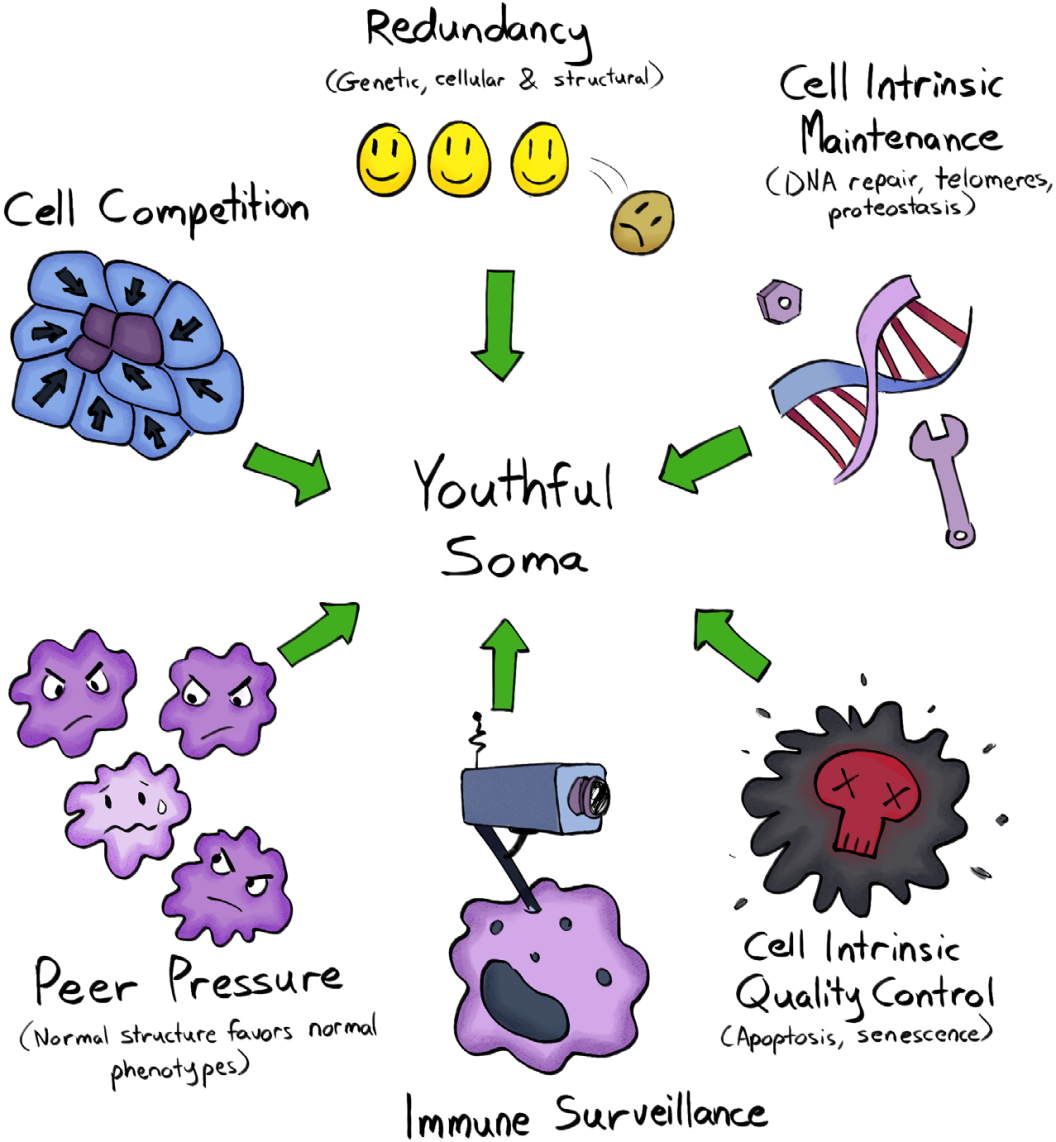
Mechanisms to maintain tissues through youth. Several maintenance programs contribute to maintain a youthful soma minimizing the propagation of non‐functional or non‐cooperating cells. These include: genetic, cellular, and structural redundancy; cell intrinsic programs such as DNA repair, apoptosis, and senescence; immune surveillance; tissue‐intrinsic mechanisms such as cell competition and the phenotype normalization of cells with malignant potential by peer pressure. [Colour figure can be viewed at wileyonlinelibrary.com]

While our cells cooperate to maintain the structure and function of tissues and organs, they are also constantly scrutinized by multifaceted quality control systems. These sophisticated mechanisms have evolved to minimize the propagation of non‐functional or non‐cooperating cells, thus preserving architectural and functional integrity while preventing oncogenesis (Fig. 3). Evolution has favored genetic programs that confer optimal cell function while being inherently tumor suppressive [61]. As a result, when cells undergo significant phenotypic change relative to wild type, they are likely to be disfavored, resulting in their elimination [90].

Some of these quality control mechanisms are cell‐intrinsic (such as apoptosis and senescence), some rely on interactions with the surrounding microenvironment (such as in cell competition), while others are mediated by tissue‐extrinsic cells and factors (as in the case of immune surveillance). While these mechanisms have evolved to be at their most efficient during our reproductive years, their effectiveness commonly declines later in life [83, 91]. Understanding how these maintenance programs are regulated and why they wane with age will help us to develop strategies to prevent or delay this decline.

### 4.1 Cell‐intrinsic programs to control mutated clones

When affected by insults or stressors, our somatic cells activate conserved mechanisms of cell‐intrinsic repair or adaptation. In most cases, minor damage is tolerated or rapidly fixed and resolved, however, more significant acute, or chronically accumulated, damage is less likely to be efficiently repaired. As a result, the affected cells undergo either controlled death (apoptosis) or enter a state of irreversible replicative arrest (senescence), thus reducing the chance that they will undergo neoplastic transformation [92] (Fig. 3).

Apoptosis enables damaged cells to be rapidly and effectively eliminated in a non‐inflammatory and non‐disruptive manner [93]. Neighboring cells normally take care of replacing the lost cell through compensatory proliferation [94]. In contrast, senescent cells lose their ability to divide while retaining their viability and functional abilities (as in wound healing [95]). Importantly, senescence prevents the clonal expansion of damaged cells and reduces the risk of their undergoing oncogenic initiation [93]. Moreover, senescent cells secrete active molecules (a state known as the senescence‐associated secretory phenotype or SASP), which signal both to the surrounding tissue and the immune system to activate tissue repair mechanisms and to promote tissue regeneration [96].

Although both apoptosis and senescence can be activated under similar stress/damage circumstances, the nature and intensity of an insult can sometimes determine which of these responses is activated [97]. For example, several DNA damaging agents induce senescence at low doses and apoptosis at higher ones [98, 99]. Other chemicals that form bulky DNA adducts exclusively trigger senescence, irrespective of the dose [100, 101]. And depending on the affected cell types, one mechanism might be favored over the other. For example, ionizing radiation (IR) normally triggers senescence in fibroblasts [100], while low IR doses induce hematopoietic stem cells (HSC) to undergo apoptosis [102]. This suggests that evolution has allowed for cells like fibroblasts, which have minimal transformation potential, to remain functional, albeit mitotically blocked; by contrast, cells with a higher risk of oncogenic transformation are more likely to be eliminated.

Although apoptosis and senescence share many molecular pathways of activation, they represent alternative fates for a cell [93]. One key factor involved in the activation of apoptosis or senescence is p53, the levels, kinetics, and transcriptional activity of which determine how a cell will respond to various stressors [103]. In particular, high p53 levels are associated with apoptosis, while attenuated p53 signaling in even severely damaged cells results in their avoiding programmed death and entering senescence [103, 104]. Conversely, p21, which is a target gene of p53 and is responsible for the initial cell cycle arrest that occurs prior to either apoptosis or senescence, seems to have an opposite role. High p21 levels are associated with the persistent cell cycle arrest that is observed in senescent cells (e.g., as occurs after treatment with a low concentration of doxorubicin), while severely damaged cells that are destined to undergo apoptosis (as occurs after high doses of doxorubicin) have low levels of p21 expression [105, 106]. Each of these two cell fates seems to counteract the other, as evidence suggests that pro‐senescence stimuli are actively anti‐apoptotic, and that senescent cells are resistant to pro‐apoptotic signals [107, 108, 109].

Interestingly, both apoptosis and senescence are key programs that are activated during embryogenesis and involved in tissue patterning (reviewed in ref. [93]). Similarly, in young individuals, these conserved mechanisms have evolved to be activated under cellular stress and, together with the immune system, they efficiently reduce cancer risk and promote tissue regeneration [110, 111]. During aging, we observe a progressive accumulation of senescent cells, partly due to a reduced ability to clear defective cells [112] and partly because of the so‐called bystander effect, whereby factors produced by the SASP can trigger senescence in neighboring cells [113]. Progressive increases in SASP signaling also contribute to the chronic low‐grade inflammatory state commonly associated with aging (called inflammaging) [77], which can be detrimental to tissue function and can in turn promote carcinogenesis through different mechanisms [114].

### 4.2 Immune surveillance

Our cells and tissues possess conserved mechanisms that signal the presence of danger (whether pathogen or damage/stress‐associated), and trigger inflammatory and immune responses that promote repair and regeneration (Fig. 3). The elimination of senescent cells is normally carried out by the immune system, both through innate and adaptive mechanisms [115]. In a recent study, during the initial stages of the mitotic block that is imposed once senescence is initiated, it was found that mouse hepatocytes were placed under immunosurveillance via the activation of p21 signaling [116]. Through the release of the chemokine, CXCL14 (a main component of the p21‐activated secretory phenotype or PASP), macrophages were promptly recruited and remained vigilant but inactive. If the hepatocytes were unable to revert to a non‐stress status within a few days (in which p21 levels were normalized), the macrophages polarized toward an M1 phenotype and cytotoxic T‐lymphocytes arrived to eliminate the target cells [116].

The clearance of senescent cells by natural killer (NK) cells has also been described after both oncogene‐ and DNA damage‐induced senescence. The overexpression of activating ligands on the surface of senescent cells [namely, MHC class I polypeptide‐related sequence A (MICA) and UL16 binding protein 2 (ILBP2)] activates NK cell receptor, NKG2D, leading to the elimination of the target cell by NK cells [117]. Another study has shown that the immunosurveillance and subsequent elimination of NRAS^G12V^‐induced pre‐malignant hepatocytes by macrophages is mediated by antigen‐specific CD4 T cells with a Th1‐phenotype [118]. Neoantigen‐specific CD8 T‐cell‐mediated elimination of cancer cells has also been documented, mainly for more advanced and aggressive tumors [119].

While the immune system is particularly successful at eliminating potentially oncogenic transformed cells throughout our reproductive years, many of its components gradually decline as we age, contributing to the emergence of what is commonly known as “immunosenescence” [77]. A marked decrease in macrophage metabolic and immunological activity, as well as a reduction in clearance and immunosurveillance capacity is commonly observed during aging [120]. Moreover, macrophages themselves become senescent in old mice, and contribute to inflammaging through SASP [121]. Adaptive immunity response is also less efficient in older individuals, owing in part to the reduced number of tissue‐resident antigen‐presenting cells and their reduced capacity to migrate and stimulate T cell activation in secondary lymphoid tissues [122]. Indeed, the decline of the adaptive immune system is mirrored by an increase in systemic inflammation as we age [77]. The activation of a proper adaptive response is also compromised by an intrinsic reduction of proteostasis and in mitochondrial activity, partly due to genetic and epigenetic alterations, which in turn, trigger senescence [123].

Finally, we also need to consider the importance of avoiding the autoimmune attack of our tissues, necessitating a balancing act that might limit the ability of the immune system to cull potentially oncogenically mutated cells. Considering that our bodies can contain well over 100 billion cells with cancer‐associated mutations that are predicted to disrupt protein function [8], many of which are likely to create new immune epitopes, we can speculate on the importance of ignoring these new epitopes for avoiding autoimmunity. Tolerance to these mutations may result from recognition of the new epitope without the co‐stimulatory signals necessary for T‐cell activation (among many other potential mechanisms). Still, this beneficial “standing down” of the immune system when faced with so many mutant clones, while allowing us to get to old age with a lowered risk of autoimmunity, might also result in pre‐existing tolerance to some mutations that eventually contribute to a full‐blown cancer.

### 4.3 Tissue‐intrinsic control mechanisms

Our tissues are finely organized such that a defined number of cells cooperate to ensure correct tissue function and structure. One evolved mechanism of tumor suppression resides in the hierarchical organization of tissue maintenance and repair systems [83]. Many of our tissues consist of a large fraction of short‐lived differentiated cells that are maintained by a limited number of stem/progenitor cells, which only proliferate to replace lost or damaged cells, as is evident in dermal, hematopoietic, and intestinal tissues, which have high turnover rates [61]. This limited number of stem and early progenitor cells represents a small target for oncogenic initiation. By contrast, mutations in differentiated cells represent a limited risk for oncogenesis as these cells have shorter lifespans and a limited ability to proliferate. For example, mutations that occur in the abundant, mature epithelial cells of the skin or intestines are likely to be eliminated and shed from the body within weeks [124].

Tissue integrity is also guaranteed by direct cell competition between homotypic cells [125] (Fig. 3). Among differentiated cells, the cells with higher relative fitness are considered to be the winners and will outcompete and actively eliminate cells with lower relative fitness (the losers) [126, 127]. While selecting the fittest cells to maximize tissue function, cell‐competition mechanisms also can prevent the clonal expansion of potentially oncogenic phenotypes. The relative fitness of our cells can be altered, compared to wild‐type, when key cellular functions are affected either by genetic or epigenetic changes [126]. For example, as shown in *Drosophila melanogaster*, cells with reduced levels of the *MYC* oncogene become losers and are selectively eliminated, whereas cells that overexpress *MYC* (albeit not excessively) behave as supercompetitors taking over surrounding wild‐type cells [128, 129, 130]. One key cellular function that determines competitiveness is protein translation, with reductions in translation leading to clonal elimination in different models from flies to mice [131]. Interestingly, in mouse HSCs, both increased and decreased translation reduces HSC fitness (leading to clonal elimination) [132]. Thus, maximal HSC fitness requires “just right” levels of translation, which will serve to facilitate the elimination of both poorly functioning and potentially transformed HSCs (as many oncogenic events are known to promote translation [133]). Cell competition mechanisms are based on the ability of our cells to sense the fitness levels of neighboring cells [126]. At the molecular level, several surface molecules are involved in this type of communication. The Flower protein is one of the most characterized sensors of cell fitness: three different Flower isoforms can signal for the survival of the winner and/or for the apoptosis of the loser, depending on their relative levels of expression on the cell surface [134, 135]. In fact, mutations that impair or promote cell competition mechanisms have been shown to reduce or increase, respectively, lifespan in flies [136]. In young and healthy tissues, cell competition represents a successful strategy to limit the survival and expansion of pre‐neoplastic cells [137, 138]. In mammalian epithelial tissues, for example, oncogenically mutated cells can be actively eliminated by neighboring healthy cells through a tissue intrinsic cell competition‐based mechanism, referred to as EDAC (epithelial defense against cancer) [139].

Another risk factor that cells can sense is loss of architectural integrity. Mutations that lead to the loss of cell polarity and tissue patterning can reduce function and trigger cancer initiation [125, 140]. Such phenotypes are normally considered losers, and the selective elimination of these cells by neighboring wild‐type counterparts has been described [140]. In the skin, for example, defective basal epidermal stem cells are actively induced by surrounding cells to downregulate the expression of collagen COL17A1, thus reducing the formation of hemidesmosomes with the basement membrane. This leads to the extrusion of damaged cells from the skin epithelium, thus preserving tissue homeostasis and preventing cancer initiation [141].

The above evidence strongly suggests that, even for a potentially oncogenic cell, the surrounding microenvironment is key to determining its fate [62, 83]. Classic studies from Beatrice Mintz (who passed away in 2022 at the age of 100) and colleagues showed that when embryonal carcinoma cells were injected into an early embryo, they contributed to many normal tissues; when transplanted into an adult mouse, they grew into cancers [27]. The tissue microenvironment is not only defined by the quality of resident cells but also by the extracellular components that help to establish that particular tissue architecture. The correct deposition and organization of extracellular matrix (ECM) components are crucial for determining cell behavior and, consequently, for correct structure and function [26]. When the ECM is disrupted, potentially tumorigenic cells have a higher chance of proliferating and progressing into cancer. As elegantly shown by Mina Bissel and colleagues, correct ECM formation can also influence the behavior of cancer cells and revert their malignant phenotype to a normal one [142]. Thus, not only does normal tissue architecture lead to the elimination of misbehaving cells, but healthy tissues also apply a form of peer pressure to normalize the behavior of cells that have malignant potential (Fig. 3). Other mechanisms are also likely to limit the phenotypic manifestation of mutated clones, including for the billions of cells that bear cancer‐associated mutations in our bodies [8]. Pathways, such as those mediated by the HSP90 chaperone, can result in phenotypic buffering, whereby the phenotypic manifestation of a mutant is limited [143, 144]. This buffering maintains a more normal phenotype and enables genotypic diversification (which is otherwise typically deleterious). When environments change, new phenotypes can then be revealed, as HSP90 functions are altered by environmental perturbations. Thus, the robustness provided by phenotypic buffering can enable evolvability [145]. These evolutionary concepts could be relevant to somatic evolution, whereby accumulating clones are kept phenotypically in check while providing adaptive solutions to changing tissue environments as we age or due to insults, such as smoking. Whether phenotypic buffering enables cells with cancer‐associated mutations to persist in our bodies, and whether oncogenic phenotypes are revealed as tissue environments change, are questions that remain to be determined.

While this review focuses on the abundant clonal expansions in our tissues that are largely driven by single mutations, we must also consider the mechanisms that help to eliminate early tumors, like carcinomas. Carcinomas *in situ* are very common in tissues like the prostate, breast, and thyroid in old adults, far outpacing the frequency of the associated cancers [146]. It is therefore important to determine what keeps these carcinomas in check. In mouse skin, clones with reduced functionality are extruded from the epithelium, as are clones with mutations in which the *HRAS* or the Wnt/β‐catenin pathway are activated, helping to preserve tissue homeostasis and the avoidance of cancer [147]. Moreover, recent studies have shown how mutant clones (such as in the Notch pathway) in the esophageal epithelium can outcompete and eliminate early tumors [148], providing additional evidence that some expanding clones could be beneficial in reducing the evolution of cancers.

**Fig. 4:**
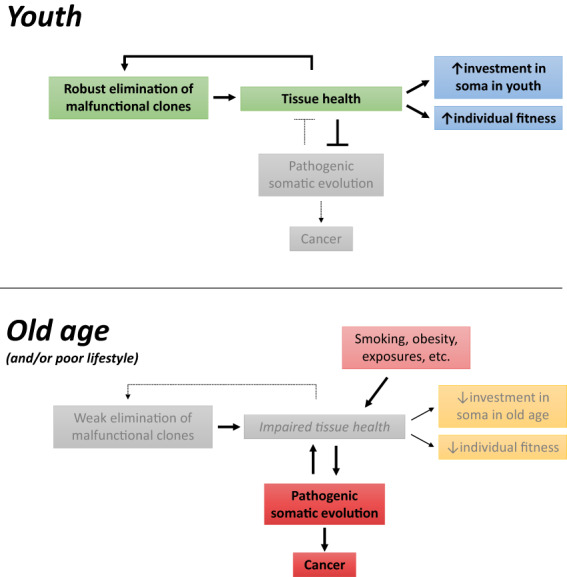
Positive reinforcement of tissue robustness in youth and its reversal in old age. In youth, poorly functioning or potentially malignant cells are eliminated from tissues, preserving tissue health and competitiveness. In old age, reduced tissue health impairs cell competition and the elimination of cells with altered functions, in a feedforward mechanism that further reduces tissue health. These mechanisms reflect investments in the soma through reproductive years and the waning of this investment in later years. [Colour figure can be viewed at wileyonlinelibrary.com]

All of these mechanisms allow our body to remain *largely* cancer‐free through our reproductive years: our cells maintain a near‐optimal fitness level and create and preserve normal tissue structure and function (Fig. 4, top). Of course, natural selection has not created perfect tissues or individuals, and there are trade‐offs associated with protective mechanisms (such as energetic costs). While a young person is much less likely to get cancer than is an old individual, such cases do occur, even for people without clear risk factors. And of course, beyond inherited factors, lifestyle factors, exposures, and even “bad luck” play important roles [149]. Moreover, as we age, a growing number of cells in our body progressively accumulate damage, which likely reduces their ability to function, compete (lower fitness), and to correctly interact and cooperate with the surrounding microenvironment. In this scenario, there is a higher chance for mutated phenotypes to confer a higher fitness, independently proliferate and initiate oncogenesis (Fig. 4, bottom).

## 5 Aging, somatic evolution, and cancer: the inexorable link

Previous authors have argued that aging is the cost of tumor suppression (e.g., [150, 151]), with the claim that preventing cancer during youth requires mechanisms, such as limited telomere maintenance, that contribute to aging phenotypes at older ages. While such antagonistic pleiotropy (Box 1) could indeed allow some tumor suppressive mechanisms to contribute to aging phenotypes, it is notable that most interventions that delay aging also reduce cancer risks (like caloric restriction or exercise), while lifestyles that reduce lifespans (like smoking) increase cancer risks [152, 153]. And, of course, the risk of many diseases, including cancers, CVD, and infections rises exponentially as humans age [2, 154]. We argue that cancers and other diseases of aging are linked in multiple ways. First, as discussed above, we experience an increased risk of disease and death late in life due to the waning of protective mechanisms at ages where we are less likely to contribute to future generations. Thus, the impact that diseases of old age have on our fitness is minimized. Second, there are common factors (like inflammation) and common conditions in addition to old age (such as smoking, exercise, diet, alcohol consumption, and obesity) that influence the risk of developing multiple diseases, from cancers to CVD.

**Fig. 5:**
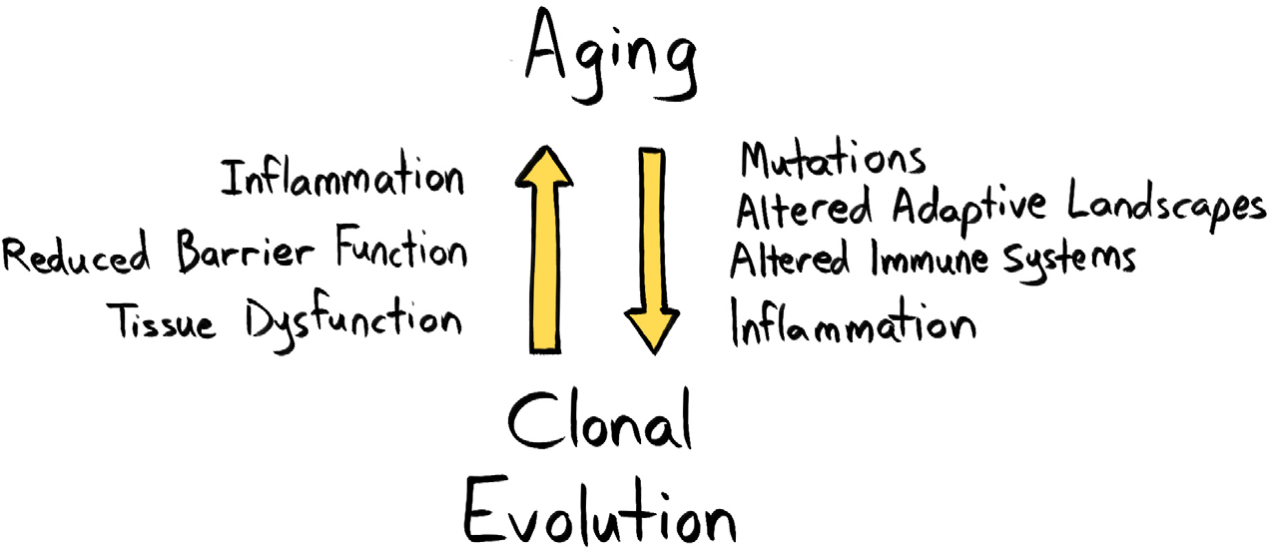
Aging promotes clonal evolution, and clonal evolution can promote aging. In this feedforward loop, the aged phenotype can promote clonal evolution through several mechanisms including the accumulation of mutations, establishing altered adaptive landscapes, and altering immune function to a more inflammatory phenotype. In turn, clonal evolution can contribute to establishing the aged phenotype, for example, promoting a pro‐inflammatory environment, reducing barrier function and, more generally, leading to tissue dysfunction. [Colour figure can be viewed at wileyonlinelibrary.com]

Could there also be common vulnerabilities in our defense or maintenance systems that lead to systemic declines in old age, with the associated multiple disease risks? We discussed above how reduced intestinal barrier function with age could represent such a tipping point, leading to systemic inflammation. The maintenance of the enormous intestinal epithelial layer in our bodies is clearly a large investment. We can surmise that such an investment would only be allocated through the years that we are most likely to contribute to subsequent generations. We also speculated that the abundance of colonic crypts with fixed cancer‐associated variants could contribute to the loss of this barrier function. Indeed, cancer‐associated mutant clones elsewhere in the body might also contribute to this loss. In fact, in mice, *Tet2* mutant hematopoiesis has been shown to reduce barrier function, leading to more systemic inflammation [34]. While we understand that aging causes oncogenesis (whether due to increasing numbers of mutations and/or to changing tissue microenvironments), “oncogenesis”—in the form of clonal expansions in apparently normal tissues—might also cause aging or at least contribute to it (Fig. 5). We have placed oncogenesis in quotes because the vast majority of clones with cancer‐associated mutations will never contribute to a malignancy. We have also discussed how mutant clones (such as those with *TET2* mutations) produce increased levels of inflammatory cytokines. Clonal hematopoiesis driven by *TET2* and other mutations is evidently associated with multiple disease risks, from cancer to CVD to chronic obstructive pulmonary disease, as reviewed above. In addition to *TET2*‐driven hematopoiesis, *NRAS* and *BCR‐ABL* oncogenic mutations promote inflammation and enhance malignant progression [66, 155, 156].

While there is a clear association between oncogene‐driven clonal expansion in the blood and disease, there is not a similarly clear association between clonal expansion in solid tissues and disease, likely due to the paucity of samples analyzed. Still, such associations are likely to be forthcoming. We can also ask why natural selection appears to have differentially disfavored such clones in different tissues. Why are such clones dominant in the esophagus, but much rarer in the liver? Does this relate to tissue turnover rates? Or could this reflect a greater fragility of some tissues, in that clonal expansions might more easily disrupt the function of these tissues, and thus natural selection has favored landscapes in these tissues that are more resistant to such expansions?

Thus, a substantial amount of research is still needed to add “mutation‐driven clonal expansions” to the list of likely suspects that cause the common patterns of multiple diseases across ages and individuals.

## 6 Conclusions and perspectives

In this review, we have discussed how natural selection has acted to prevent the expansion of mutation‐bearing clones that could lead to cancer or otherwise disrupt tissue function, and how this pressure wanes at older ages when our chances of reproducing are reduced. We have also reviewed some of the mechanisms that have evolved to eliminate clones with mutations that disrupt cell function, which act to maintain tissue health (youthful fitness) and to impede cancer development. Emerging but still incomplete evidence points to a convergence between evolutionary theories of aging and cancer, with common mechanisms preventing aging and cancer during youth and then enabling aging‐associated tissue decline and cancer in old age. As discussed here, evidence also highlights connections between tissue decline and somatic evolution; tissue landscapes altered by old age or other insults can promote somatic evolution, which can sometimes lead to cancers. Importantly, expanding and mutation‐bearing clones that disrupt key cell fate pathways are also likely to reduce tissue function independently of malignant progression. Thus, aging promotes somatic evolution, which itself might also promote aging, establishing a feedforward loop that might contribute to our accelerating decline at older ages. Finally, while incomplete, a picture is emerging in which most mutations seen in clonal expansions in human tissues, particularly in younger individuals, are rarely observed in human cancers, even though many are cataloged in the Cancer Gene Consensus database as having been observed at least once in a cancer [8]. In contrast, the mutations most commonly observed in human cancers are rarely observed in normal human tissues, particularly in younger individuals [8]. Thus, the selective pressure to limit the positive selection of a mutation appears to be proportional to its malignant potential (and perhaps also to its tissue‐damaging potential), consistent with predictions from theoretical and computational modeling [157]. Natural selection has not acted to prevent all somatic evolution, just the clonal expansions that could limit individual fitness.

In all, we can begin to recognize that there are inexorable links between aging, somatic evolution, cancers, and other diseases, both through functional links within our tissues and by the converging forces of natural selection across millennia. This new perspective should help to guide strategies for the prevention and treatment of diseases of aging, such as by engendering tissue landscapes that promote benign clonal evolution while impeding damaging clonal expansions.

## Conflict of interest

The authors declare no conflict of interest.

## Author contributions

JD and FM conceived, designed, and wrote the manuscript.
